# The Entner-Doudoroff Pathway Contributes to Glycogen Breakdown During High to Low CO_2_ Shifts in the Cyanobacterium *Synechocystis* sp. PCC 6803

**DOI:** 10.3389/fpls.2021.787943

**Published:** 2021-12-09

**Authors:** Stefan Lucius, Alexander Makowka, Klaudia Michl, Kirstin Gutekunst, Martin Hagemann

**Affiliations:** ^1^Department of Plant Physiology, Institute of Biosciences, University of Rostock, Rostock, Germany; ^2^Department of Biology, Botanical Institute, Christian-Albrechts-University, Kiel, Germany; ^3^Department of Molecular Plant Physiology, Bioenergetics in Photoautotrophs, University of Kassel, Kassel, Germany; ^4^Interdisciplinary Faculty, Department Life, Light and Matter, University of Rostock, Rostock, Germany

**Keywords:** CO_2_ acclimation, glycolytic pathways, metabolome, mutant, sugar catabolism

## Abstract

Cyanobacteria perform plant-like oxygenic photosynthesis to convert inorganic carbon into organic compounds and can also use internal carbohydrate reserves under specific conditions. A mutant collection with defects in different routes for sugar catabolism was studied to analyze which of them is preferentially used to degrade glycogen reserves in light-exposed cells of *Synechocystis* sp. PCC 6803 shifted from high to low CO_2_ conditions. Mutants defective in the glycolytic Embden–Meyerhof–Parnas pathway or in the oxidative pentose-phosphate (OPP) pathway showed glycogen levels similar to wild type under high CO_2_ (HC) conditions and were able to degrade it similarly after shifts to low CO_2_ (LC) conditions. In contrast, the mutant Δ*eda*, which is defective in the glycolytic Entner-Doudoroff (ED) pathway, accumulated elevated glycogen levels under HC that were more slowly consumed during the LC shift. In consequence, the mutant Δ*eda* showed a lowered ability to respond to the inorganic carbon shifts, displayed a pronounced lack in the reactivation of growth when brought back to HC, and differed significantly in its metabolite composition. Particularly, Δ*eda* accumulated enhanced levels of proline, which is a well-known metabolite to maintain redox balances via NADPH levels in many organisms under stress conditions. We suggest that deletion of *eda* might promote the utilization of the OPP shunt that dramatically enhance NADPH levels. Collectively, the results point at a major regulatory contribution of the ED pathway for the mobilization of glycogen reserves during rapid acclimation to fluctuating CO_2_ conditions.

## Introduction

Cyanobacteria evolved oxygenic photosynthesis approximately 2.7 billion years ago (Hohmann-Marriott and Blankenship, 2011). By this process, they can efficiently convert inorganic carbon (Ci), either as CO_2_ or bicarbonate into organic material at the expense of light energy. This capability makes cyanobacteria an interesting chassis for the CO_2_-neutral production of organic fuels or feedstock (e.g., Hagemann and Hess, 2018). The Calvin-Benson-Bassham (CBB) cycle is the main Ci incorporating route with ribulose 1,5-bisphosphate carboxylase/oxygenase (RubisCO) as key enzyme, which incorporates either CO_2_ into ribulose 1,5-bisphosphate (RuBP) leading to two molecules 3-phosphoglycerate (3PGA) or O_2_ into RuBP resulting in the appearance of 3PGA and 2-phosphoglycolate (2PG). The latter intermediate turned out to be toxic for the CBB cycle and associated metabolic pathways (e.g., Flügel et al., 2017) and must be efficiently recycled via the photorespiratory 2PG metabolism, which likely evolved in parallel with oxygenic photosynthesis in ancient cyanobacteria (Eisenhut et al., 2008a).

The available amount of Ci for photosynthesis changed in different time scales in the environment of cyanobacteria. In the long term, it dramatically decreased to the present low CO_2_ content of about 0.04% in the Earth’s atmosphere mainly due to the photosynthetic activities of cyanobacteria, algae and plants. In parallel, the amount of O_2_ rose to 21%. This gas ratio appeared to be problematic for RubisCO, because it has a rather low affinity and specificity for CO_2_ (Tcherkez et al., 2006). Especially in aquatic habitats, the Ci levels are also fluctuating in short time scales depending on the pH, salinity and temperature influencing the solubility of CO_2_ and its conversion into bicarbonate. To respond to limiting Ci conditions, cyanobacteria evolved an inorganic carbon-concentrating mechanism (CCM), which enables them to accumulate CO_2_ in the vicinity of RubisCO, permitting the enzyme to work under saturated CO_2_ conditions thereby suppressing the oxygenase reaction (Kaplan et al., 2008). The CCM activity in model strains such as *Synechocystis* sp. PCC 6803 (hereafter *Synechocystis*) is mainly regulated at the transcriptional level due to the action of several regulatory proteins such as NdhR, CmpR, CyAbrB2, and SbtB (reviewed in Hagemann et al., 2021).

In addition to the transcriptional changes, the shift from high to low Ci conditions had pronounced effects on the metabolic composition of cyanobacteria (Eisenhut et al., 2008b; Schwarz et al., 2011). In the case of *Synechocystis*, these alterations seem to be mainly regulated on the biochemical level (Jablonsky et al., 2016), because the abundances of transcripts and proteins for almost all enzymes involved in primary carbon metabolism remained unchanged under different Ci conditions (Klähn et al., 2015; Spät et al., 2021). A defined metabolic signature was observed in *Synechocystis* cells shifted from high Ci (HC, 5% CO_2_) to low Ci (LC, ambient air of 0.04% CO_2_), which is at least partly conserved in Arabidopsis plants (Orf et al., 2016). This metabolic change indicates that the carbon export from the CBB cycle into lower glycolysis is stimulated under LC conditions, which might involve the newly identified regulator protein of phosphoglycerate mutase PirC (Orthwein et al., 2021). Furthermore, HC-grown *Synechocystis* cells accumulate high glycogen levels. Upon shifts to LC conditions glycogen becomes degraded and long-term LC-acclimated cells are virtually free of glycogen reserves (Eisenhut et al., 2007).

Hence, different Ci conditions do not only affect carbon anabolism via the CCM and the CBB cycle but have marked influences on carbon partitioning and glycogen accumulation or degradation. Basically, four different routes have been identified, which are involved in the metabolism of glucose released from glycogen breakdown in *Synechocystis* and other cyanobacteria. In darkened cells, the oxidative pentose-phosphate (OPP) pathway has been shown to be mainly responsible for the utilization of accumulated glycogen reserves (Pelroy et al., 1972; Makowka et al., 2020). Two different glycolytic routes, the Embden–Meyerhof–Parnas (EMP) and the Entner-Doudoroff (ED) pathway are also active in cyanobacteria (Chen et al., 2016). Recently it has been shown that the ED pathway, which shows the lowest overlap with the CBB cycle, plays the main role for carbon catabolism in the light during recovery from long-term nitrogen limitation (Doello et al., 2018). Furthermore, these pathways also support the reactivation of the CBB cycle during transition from darkness into light, however, under this condition they transiently do not operate at their full length but instead probably form short shunts (Makowka et al., 2020). As fourth route, the phosphoketolase pathway can also contribute to glucose catabolism under specific conditions in *Synechocystis* (Bachhar and Jablonsky, 2020; Chuang and Liao, 2021).

**FIGURE 1:**
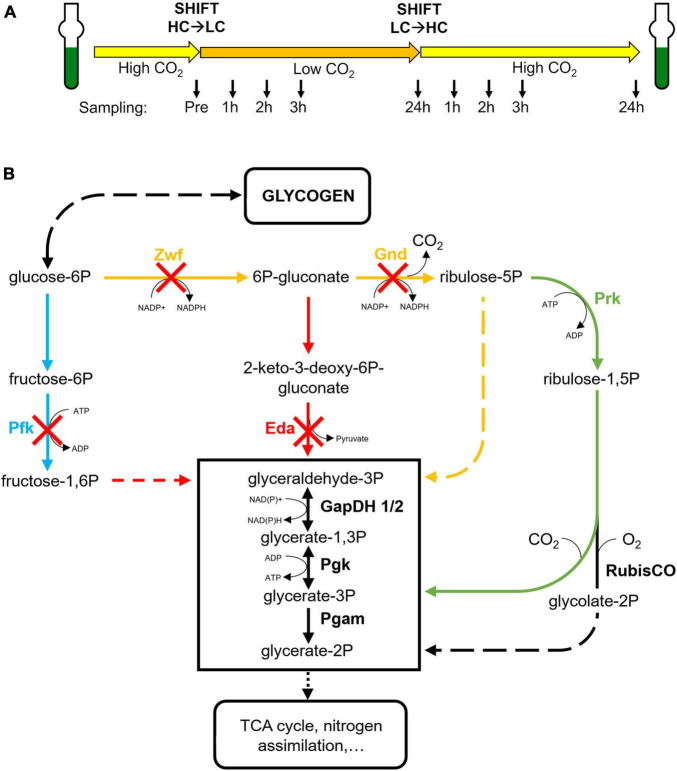
Experimental design and affected enzymes in the studied strains. **(A)** All strains were pre-cultured under high CO_2_ (5%, HC) and then shifted for 24 h into low CO_2_ (ambient air, 0.04%, LC) and afterwards again into HC conditions. At defined time points (indicated by arrows) samples were taken for glycogen and metabolome analyses. **(B)** Schematic display of the primary carbon metabolism in cyanobacteria with the CBB cycle, the oxidative pentose-phosphate (OPP) pathway, the glycolytic Embden–Meyerhof–Parnas (EMP) and the Entner-Doudoroff (ED) pathways. The absence of specific enzymes in the mutants Δ*pfk*, Δ*zwf*, Δ*gnd*, and Δ*eda* are indicated by red crosses. (blue – EMP pathway, red – ED pathway, yellow – OPP pathway, green – CBB cycle).

In the present study, exclusively the roles of three routes, the OPP, EMP and ED pathways were characterized. We analyzed which of these glucose catabolic routes is mainly involved in the mobilization of glycogen reserves during HC to LC shifts. For this purpose, the response to fluctuating Ci conditions was compared between the *Synechocystis* wild type (WT) and a set of mutants defective in the OPP, the glycolytic EMP or ED pathways (Figure 1). Our results indicate a major regulatory impact of the glycolytic ED pathway on glycogen accumulation in the light and its mobilization during HC to LC shifts, which is crucial for a rapid acclimation to fluctuating CO_2_ conditions.

## Materials and Methods

### Strains and *Synechocystis* Mutants

All analyzed wild-type and mutant strains of *Synechocystis* sp. PCC 6803 used in this study are described by Chen et al. (2016).

### Shift Experiments

Cells of the *Synechocystis* WT and the mutants were pre-cultivated in batch cultures under high Ci-conditions (5% CO_2_, HC) in buffered BG-11 medium (Rippka et al., 1979; TES pH 8.0) containing the respective antibiotics at 30°C and 100 μmol photons m^–2^ s^–1^ in glass tubes until the cell suspension reached the desired OD at 750 nm (OD_750_) of 0.8 to 1.0. After 24 h of acclimation the *Synechocystis* cultures were diluted to OD_750_ = 0.8 with fresh BG-11 (pH 8.0). After cultivation for another hour, samples for LC-MS and glycogen analysis were harvested and OD_750_ was determined. To shift cultures from HC to low CO_2_ (LC, 0.04% CO_2_ in ambient air) conditions, cells were then centrifuged and resuspended in identical volume of BG-11 (pH 7.0) before continuing bubbling cultivation with ambient air. After 24 h, cultures were shifted back from LC to HC conditions by changing the aeration supply back to air containing 5% CO_2_. Samples were taken one, two, three and 24 h after each CO_2_ shift and OD_750_ was measured as proxy for growth and biomass (see Figure 1A).

### Glycogen Quantification

Cellular glycogen content determination was modified after Gründel et al. (2012), as described in Makowka et al. (2020). During the CO_2_ shift experiments, 5 mL of cells were harvested in duplicates and pelleted by centrifugation. The pellet was resuspended in 300 μL 30% (w/v) KOH and cells were incubated at 95°C for 2 h. After addition of 900 μL ethanol, samples were incubated at −20°C over night. Samples were then centrifuged (10 min, 10000 g, 4°C) for glycogen precipitation. Pellets were washed with 1 mL absolute and 1 mL 70% ethanol and then dried at 50°C. These pellets were resuspended in 200 μL sodium acetate buffer (100 mM, pH 4.5) containing 21 U amyloglucosidase and incubated at 60°C for 90 min. After centrifugation (10 min, 10000 g, RT), the released glucose was determined in the supernatants using o-toluidine reagent. Glucose contents were calculated using a glucose calibration curve.

### Metabolite Analysis

During the CO_2_ shift experiments cells were harvested in duplicates of 5 mL at each respective time point on nitrocellulose filters (25 mm, Porafil, Macherey-Nagel) and resuspended in 1 mL ethanol (80%, HPLC grade, Roth, Germany). The metabolites were later normalized according to their respective OD750nm and sample volumes. Low molecular mass compounds were extracted from the cells with ethanol at 65°C for 2 h. One microgram of carnitine was added per sample as an internal standard. After centrifugation, the supernatants were collected and freeze-dried. The dry extracts were dissolved in 800 μl MS-grade water and filtered through 0.2 μm filters (Omnifix^®^-F, Braun, Germany). The cleared supernatants were analysed using the high-performance liquid chromatograph mass spectrometer system (LCMS-8050, Shimadzu, Japan). In brief, 1 μl of each extract was separated on a pentafluorophenylpropyl (PFPP) column (Supelco Discovery HS FS, 3 μm, 150 x 2.1 mm) with a mobile phase containing 0.1% formic acid. The compounds were eluted at a rate of 0.25 ml min^–1^ using the following gradient: 1 min 0.1% formic acid, 95% distilled water, 5% acetonitrile, within 15 min linear gradient to 0.1% formic acid, 5% distilled water, 95% acetonitrile, 10 min 0.1% formic acid, 5% distilled water, 95% acetonitrile. Aliquots were continuously injected in the MS/MS part and ionized via electrospray ionization (ESI). The compounds were identified and quantified using the multiple reaction monitoring (MRM) values given in the LC-MS/MS method package and the LabSolutions software package (Shimadzu, Japan). The metabolites were determined as relative metabolite abundances, which were calculated by normalization of signal intensity to that of the internal standard carnitine and optical density per sampling volume.

### Data Evaluation and Statistical Analysis

The experiments were repeated at least three times with independent cultivations. Data evaluation for LC-MS was done by first calculating the averages of absolute metabolite contents per sampling point within each biological replicate and then calculating the fold change to the respective WT HC pre-shift (set to 1) data. Final metabolite values were obtained by then calculating the average of all three biological replicates’ fold change data. The growth and glycogen datasets are representative of three biological replicates. A Student’s *t*-test with significance level of 5% (*p* ≤ 0.05) was performed. A significant deviation of every single data point of a mutant strain compared to respective WT data is marked by an asterisk. Heat map data were calculated for each metabolite as fold changes (log_10_) to HC pre-shift values (set to 1) in WT cells. Heat maps were created in program MultiExperiment Viewer (TM4 MeV). Principal component analyses (PCAs) were performed using the program Minitab^®^ (version 17.1.0., Minitab Inc.) with metabolite fold change values compared to the respective WT HC pre-shift data (set to 1). The first two principal components cover 52.5% of the total variance of the dataset data set, as calculated by the software during data analysis.

## Results

To analyze which glucose catabolic route is mainly responsible for the mobilization of glycogen reserves during HC to LC shifts, cells of the *Synechocystis* WT and a set of mutants defective in specific key enzymes of the OPP, the glycolytic EMP or ED pathways were compared under different CO_2_ availability. Specifically, the previously described mutants Δ*pfk* [EMP pathway blocked due to the absence of two isoforms of phosphofructokinase (Pfk)], Δ*zwf* [OPP and ED pathways blocked due to the absence of glucose 6-phosphate dehydrogenase (Zwf)], Δ*gnd* [OPP pathway blocked due to the absence of 6-phosphogluconate dehydrogenase (Gnd)], and Δ*eda* [ED pathway blocked due to the absence of 2-dehydro-3-deoxyphosphogluconate aldolase (Eda)] were used (Chen et al., 2016) (Figure 1B). All strains were first acclimated for at least one week to HC conditions (5% CO_2_), then shifted to LC (ambient air with 0.04% CO_2_) for 24 h and subsequently brought back to HC for 24 h (HC to LC to HC shifts; Figure 1A). At different time points samples were taken for glycogen and metabolome analysis, while the growth of the cultures was monitored via measurements of optical densities at 750 nm (OD_750_).

### Alterations of Glycogen Levels in Strains During HC-LC-HC Shifts

**FIGURE 2:**
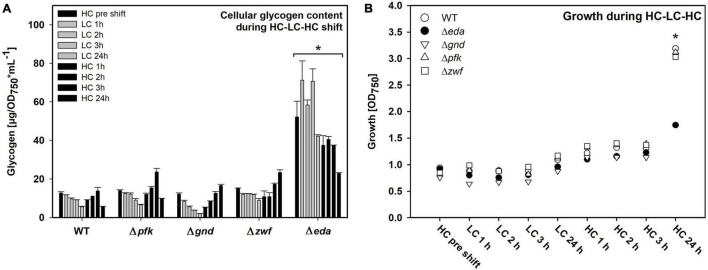
Glycogen pools and growth of the different strains during HC-LC-HC shifts. **(A)** Glycogen amounts were determined enzymatically. Asterisk indicates significant differences (*p* ≤ 0.05) of every single Δ*eda* value compared with respective wild-type (WT) data. **(B)** Growth of the different strains during HC-LC-HC shifts. The optical density at 750 nm (OD_750_) was measured at every sampling point during the shift experiment and is used as proxy for growth (*n* = 3). Significant difference (*p* ≤ 0.05) between Δ*eda* and all other strains are marked by an asterisk (representative for three biological replicates).

Different patterns of changes in the glycogen pool were observed. As expected, the WT cells reduced the initial HC level of glycogen during the 24 h LC period and restored it in the following HC period (Figure 2A). This glycogen level pattern was principally also found in the mutants Δ*pfk*, Δ*zwf* and Δ*gnd*. However, the Δ*eda* mutant showed marked differences. Cells of this strain accumulated significantly higher glycogen levels under HC conditions, which were not mobilized to a significant extent during the first hours of the LC period (Figure 2A). Only at the end of the LC period the glycogen level decreased. Furthermore, the somehow reduced glycogen pool was not recovered in the mutant Δ*eda* during the next HC period as in the other strains.

All mutants decreased growth to almost zero during the 24 h LC period (Figure 2B). Growth resumed stepwise during the following HC period for WT and mutants Δ*pfk*, Δ*zwf* and Δ*gnd*. However, the mutant Δ*eda* showed a pronounced lack in the reactivation of growth under the HC conditions. These results clearly show that absence of Eda has a strong impact on glycogen accumulation and remobilization in *Synechocystis* during HC to LC shift, and that this capability is essential for a rapid acclimation to fluctuating CO_2_ conditions.

### Global Changes of the Metabolome in Strains During HC-LC-HC Shifts

**FIGURE 3:**
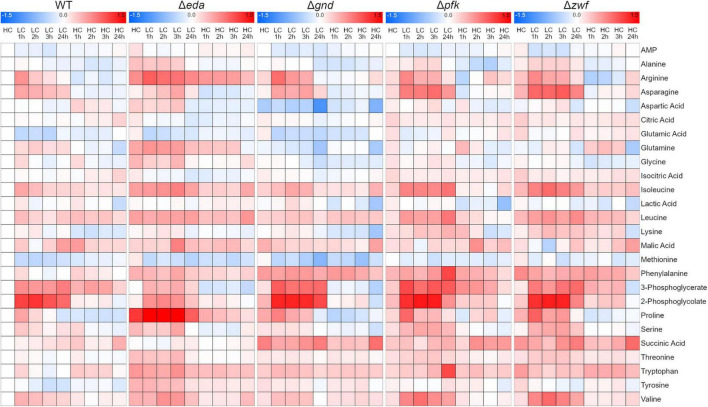
Heat maps displaying the metabolic changes in different strains during HC-LC-HC shifts. The relative changes in the amounts of 26 metabolites are shown. The metabolite level in wild-type cells (WT) in the initial HC phase was set to one. All other metabolite values in the different strains at each sampling point are shown as fold changes (log_10_ values) related to HC value in WT cells (red – increases, blue – decreases) (*n* = 3).

Next, we aimed to analyze whether the mutation of different glucose metabolizing pathways is also impacting the general carbon and nitrogen metabolism. A targeted metabolome approach was used that permitted to quantify 26 metabolites in three independent experiments. The quantified compounds include mostly amino and organic acids, whereas the phosphorylated intermediates up- and downstream of the mutations could not be detected by this method. The metabolic analysis shows that many metabolites responded to the HC-LC-HC shift conditions in these strains (the entire dataset is displayed in the Supplementary Table 1). These changes are illustrated in the corresponding heat map showing that selected metabolites respond differentially to the HC-LC-HC shifts in the different strains, while many metabolites show similar patterns in the mutants and the WT (Figure 3). For example, the RubisCO oxygenase reaction product 2-phosphoglycolate (2PG) showed the expected rapid accumulation in the WT and all mutants after LC shift. As expected, 2PG levels resumed in the subsequent HC period, whereas the RubisCO carboxylation product 3-phosphoglycerate (3PGA) showed lower increases after the LC shift. Glutamate and some other amino acids such as methionine showed an opposite behavior (Figure 3).

**FIGURE 4:**
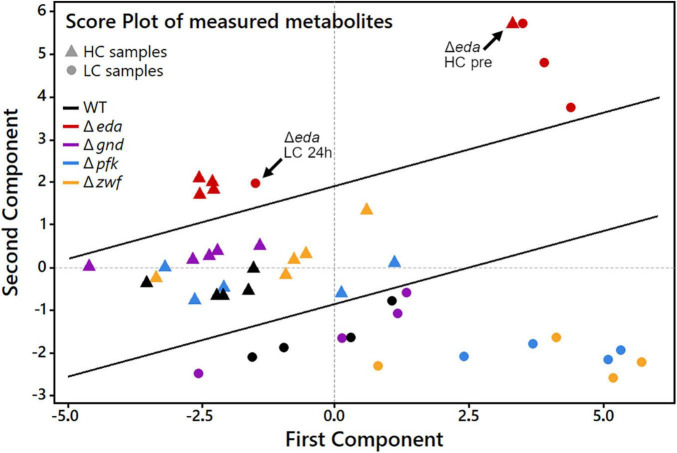
Principal component analysis (PCA) score plot to analyze patterns in samples by genotype and CO_2_ treatment during HC-LC-HC shifts. Every data point represents one sample as a combination of genotype and one of the nine sampling time points during the HC-LC-HC shift experiment. HC and LC samples, regardless of sampling time, are represented only by the same respective genotype symbol for clarity reasons. Inserted black lines mark the separation of LC, HC and Δ*eda* samples, respectively.

The global metabolic changes in the different strains under changing Ci conditions were further analyzed using principal component analysis (PCA). This analytical tool projects a high-dimensional dataset into a low-dimensional space. Multiple measurement parameters of an analysis are transformed into few principal components in a new orthogonal coordinate system. The thus obtained PCA score plot displays similarities regarding all quantified metabolites at each sampling time point during the HC-LC-HC shift in the respective genotypes (Figure 4). The multidimensional dataset in which every measured metabolite represents one dimension per sample has therefore been reduced to a two-dimensional score plot with only two principal components. These two components are the most important for data interpretation as they explain 52.5% of the total measured variance between all data, which can be considered as the most significant contribution to their separation. Every data point in the score plot thus represents one experimental sample defined by genotype, CO_2_ treatment and sampling time that is plotted according to its specific metabolic composition measured in the LC-MS analysis. As observed before for the glycogen accumulation patterns, the metabolic changes in the mutant Δ*eda* showed clear differences compared to the WT and the other mutants. The metabolite composition in Δ*eda* is always above all other strains in the second dimension (Figure 4). It shows higher first component values regarding the metabolite composition in the initial HC state and the first LC samples. This separation indicates that the increased glycogen level and its slow consumption in cells of mutant Δ*eda* induces a general different metabolic composition and a delayed acclimation to LC conditions, respectively. Among the other genotypes, a clear separation of metabolic composition is found under HC versus LC conditions, mainly defined by the second component. Apart from Δ*eda*, HC metabolic compositions in the mutants scatter together with WT in the first component and are generally shifted to lower second component values for all LC samples. Among these LC metabolic compositions, WT and mutant Δ*gnd* are very close to each other, while it differs slightly more between WT and mutants Δ*pfk* and Δ*zwf* (Figure 4). Generally, the first principal component is mostly sufficient to distinguish HC and LC treatments while the second component mainly reveals the difference of Δ*eda* and all other strains.

**FIGURE 5:**
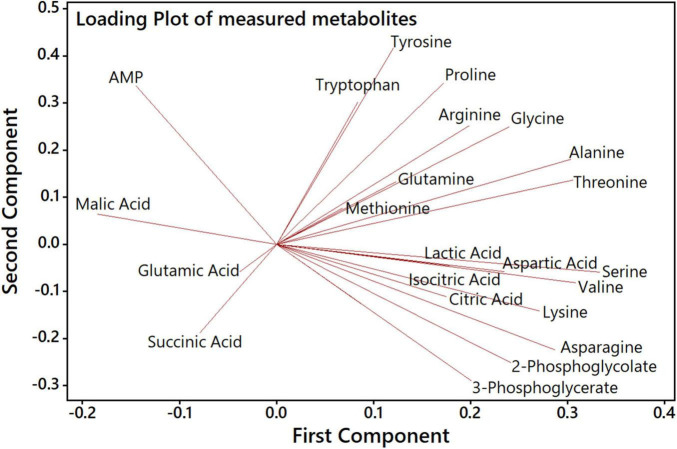
PCA loading plot to analyze the influence of specific metabolites on the first and second component. Each quantified metabolite is represented by a vector, whereby length, angle and direction indicate the contribution of that metabolite to the first and second component.

Next, we aimed to analyze how far specific metabolites are determining the separation of genotypes and CO_2_ treatments in the PCA shown in Figure 4. The accompanying PCA loading plot (Figure 5) displays, which of the measured metabolites has the highest influence on defining the two principal components. The loading plot points out that the metabolites 3PGA and 2PG show the highest contribution to the first component and thus might be mainly responsible for the clear separation of most HC from LC samples in the score plot. The low angles between the respective vectors indicate a high positive correlation of these metabolites in the dataset. Vectors for proline, arginine, and glutamine point at the direction of the observed Δ*eda* LC cluster in the score plot and therefore might represent metabolites responsible for the separation of these data. Their almost 180° angles toward glutamic acid and succinic acid vectors suggest a strong negative correlation between their metabolic behavior during the HC-LC-HC shift (Figure 5). Hence, these metabolites deserved closer inspections, since their changes could be related to alterations in carbon flux or nitrogen assimilation due to the different mutations.

### Specific Changes of the Metabolome in Strains During HC-LC-HC Shifts

**FIGURE 6:**
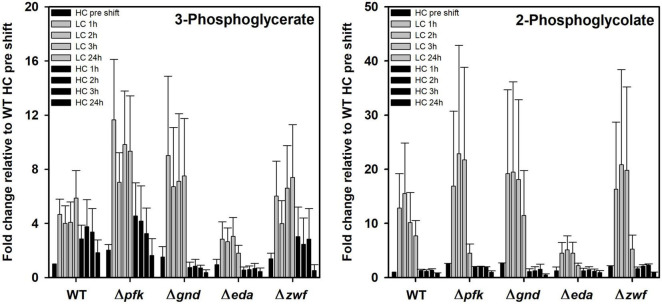
Changes in RubisCO reaction products in different strains during HC-LC-HC shifts. Alterations in the contents of 3-phosphoglycerate and 2-phosphoglycolate are given as fold changes relative to wild-type values (WT, HC pre shift was set to 1) under HC conditions at the beginning of the HC-LC-HC shift experiment. Mean values and standard deviations of fold changes are displayed from all three independent experiments.

The previous PCA indicates that specific metabolites show significant differences in their response to different Ci conditions in the mutants compared to WT. Marked changes were observed for the RubisCO reaction products 3PGA and 2PG (Figure 6). 3PGA increased approximately 4-fold after the shift of HC-acclimated cells to LC, while the 3PGA contents returned to lowered levels during the subsequent HC period. The 3PGA increase under lowered CO_2_ availability rather points at a diminished metabolism of this compound in the CBB cycle than at an increased RubisCO carboxylation activity. Obviously, readjusting the CBB cycle activity needs more time during LC acclimation. Basically, similar patterns were observed in the different mutant strains, especially in the mutant Δ*zwf*. However, the extent of changes differed in the other mutants. 3PGA became more strongly accumulated in the mutants Δ*pfk* and Δ*gnd*. In the mutant Δ*gnd* it declined immediately to the initial HC levels when cells were again shifted to HC (Figure 6). Compared to all other strains, the mutant Δ*eda* showed the smallest increase of 3PGA in LC-shifted cells. Similar observations were made of the oxygenase reaction product 2PG, which, however, showed a higher stimulation in LC-shifted cells compared to the initial HC level that indicates the expected higher stimulation of the oxygenation than the carboxylation activity of RubisCO under this condition. Again, the mutant Δ*eda* showed a significantly smaller increase than WT, while all other mutants have the tendency to increase 2PG to a higher extent than WT. In all strains, the 2PG level returned immediately to the low, initial HC level after the retransfer into HC (Figure 6).

**FIGURE 7:**
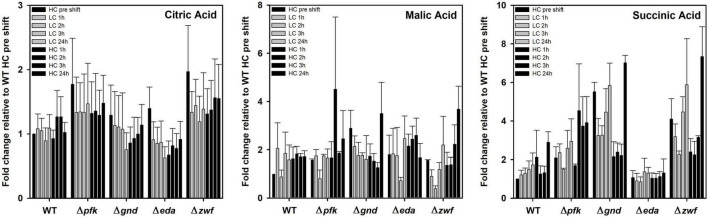
Changes in TCA cycle intermediates in different strains during HC-LC-HC shifts. Alterations in the contents of citric acid, malic acid and succinic acid are given as fold changes relative to wild-type values (WT, HC pre shift was set to 1) under HC conditions at the beginning of the HC-LC-HC shift experiment. Mean values and standard deviations of fold changes are displayed from all three independent experiments.

Some organic acids, especially intermediates of the TCA cycle showed a different response in the mutants compared to WT as well under different Ci conditions. Citric acid is almost without changes in WT and mutants Δ*pfk* and Δ*zwf.* However, its content decreased 24 h after LC shift in the mutants Δ*gnd* and Δ*eda*, thereafter it returned stepwise to the initial level in the subsequent HC period (Figure 7). Succinate was stepwise accumulated in the WT after LC shift, but it remained unchanged under different Ci regimes in mutant Δ*eda*. The mutants Δ*zwf* and Δ*gnd* accumulated more succinate under HC conditions and do not show the increase under LC conditions (Figure 7). In contrast to succinate, no large deviations were seen in the pattern of malate. Its level declined in most strains 2-3 h after LC shift, but this drop was not observed for the mutant Δ*gnd* (Figure 7).

**FIGURE 8:**
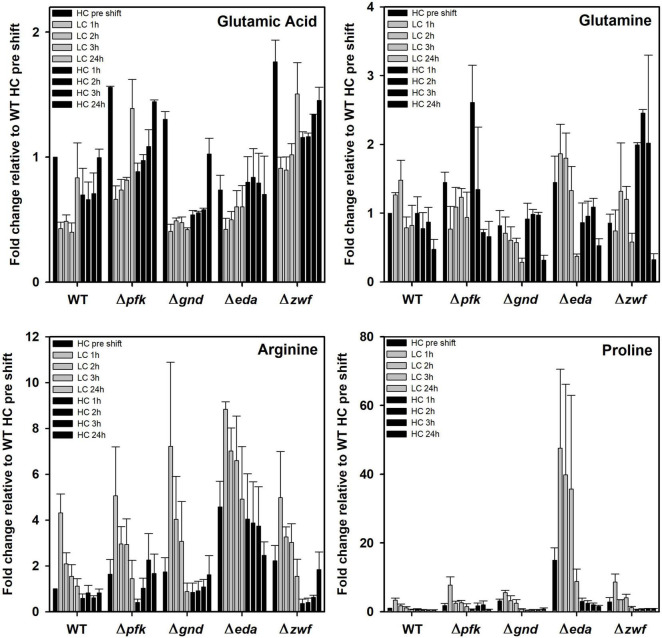
Changes in selected amino acids in different strains during HC-LC-HC shifts. Alterations in the contents of glutamic acid, glutamine, arginine, and proline are given as fold changes relative to wild-type values (WT, HC pre shift was set to 1) under HC conditions at the beginning of the HC-LC-HC shift experiment. Mean values and standard deviations of fold changes are displayed from all three independent experiments.

Finally, some amino acids showed marked deviations between WT and mutants under the different Ci conditions. In this regard it is remarkable to see the very pronounced accumulation of proline in the mutant Δ*eda* (Figure 8). Its content is about 10 times higher in HC-cultivated cells of this mutant cells compared to all other strains and increased to 40-fold higher levels in LC-shifted cells. The LC-stimulated accumulation of proline was also seen in the other strains, however, to a much smaller extent. Proline is a well-known regulatory metabolite that maintains redox balances via the modulation of NADPH levels in bacteria, animals and plants during many stress conditions (Fichman et al., 2015; Zhang and Becker, 2015). Its reversible biosynthesis from glutamate requires two NADPH. Hence, proline synthesis and oxidation can either protect cells from redox stress due to the overproduction of reactive oxygen species (ROS) or alternatively support ROS formation to induce programmed cell death. The accumulation of proline in Δ*eda* thus points at much larger perturbations in NADPH levels in this mutant when shifted from HC into LC conditions. After the subsequent HC shift, proline returned to the initial levels in all strains.

**FIGURE 9:**
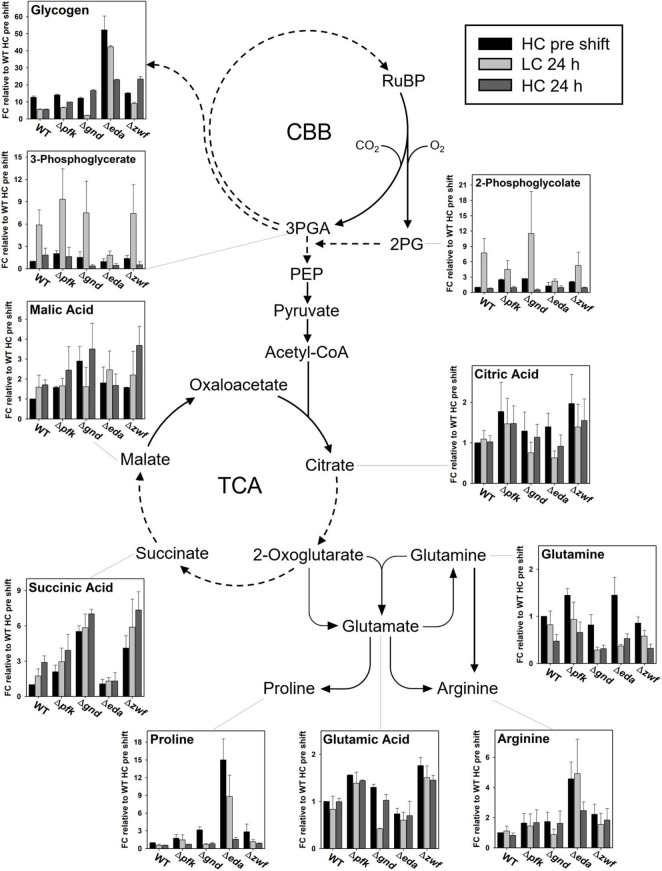
Steady states of selected metabolites in the context of entire metabolism. Dashed arrows indicate that more than one enzymatic reaction is required to catalyze this step. (FC – fold change).

In addition to proline, arginine accumulates also to 4-fold higher levels in mutant Δ*eda* compared to WT. Arginine increased in all strains after the LC shift, where the second highest stimulation was observed in mutant Δ*gnd* that reached almost the levels of Δ*eda* (Figure 8). Glutamate represents by far the most abundant amino acid in *Synechocystis* and serves as amino donor in most transaminase reactions. Together with glutamine, it is part of the GS/GOGAT cycle for ammonia assimilation in cyanobacteria as in plants. Higher glutamate amounts than in WT were observed in the mutants Δ*pfk*, Δ*gnd*, and Δ*zwf*, but not in Δ*eda* in the HC-acclimated cells prior to the shifts (Figure 8). Its amount decreased in all cases during the first hours in LC-shifted cells, but returned to the initial level after 24 h LC with the exception of mutant Δ*gnd*. In the latter mutant the initial glutamate level became only restored after 24 h growth under HC conditions. Again, only in the case of Δ*gnd* stronger deviations were found for glutamine compared to WT (Figure 8). This mutant accumulated less glutamine and showed the strongest decline in its content after the LC shift. It is also interesting to note that glutamine became strongly re-accumulated in cells of the mutants Δ*pfk* and Δ*zwf* after the subsequent HC shift. To support the understanding of the metabolic changes, selected metabolites are displayed in the context of entire carbon metabolism for the initial HC and end of the subsequent LC or HC periods in Figure 9.

## Discussion

Glycogen is the main carbon storage in cyanobacteria, which is synthesized via photosynthesis and gluconeogenesis under Ci excess conditions during the day and becomes consumed via the OPP pathway and respiration to produce carbon precursors and energy at night. In addition to the diurnal glycogen cycle, it has been shown that glycogen levels are also fluctuating in *Synechocystis* in constant light, when carbon or nitrogen availability is changing (e.g., Eisenhut et al., 2007; Doello et al., 2018). In contrast to other glycolytic routes, intermediates of the ED pathway are not overlapping with the CBB cycle and pyruvate can be provided directly without the need for lower glycolysis (Chen et al., 2016). This makes the ED pathway particularly suitable for carbon catabolism in the light without interfering with photosynthetic CO_2_ fixation. The results from the present study point at a major, but most likely regulatory contribution of the ED shunt for the mobilization of glycogen reserves in the light, which contributes to the rapid acclimation to fluctuating CO_2_ conditions. This main conclusion is based on the clearly elevated glycogen pool and its delayed mobilization upon shift to LC in combination with dramatically elevated levels of proline in the Δ*eda* mutant. Similar observations, i.e., a decelerated glycogen consumption in the presence of an active CBB cycle were made, when the *Synechocystis* mutant Δ*eda* was grown at constant LC conditions in the light (Makowka et al., 2020).

Obviously, the presence of Eda has a great impact on the entire carbon and associated nitrogen metabolism and supports a rapid reactivation of growth after periods of LC when HC became newly available. This finding was not expected, because the strong decrease in glycogen pools after long-term shifts into LC conditions has been interpreted such that in the presence of limited Ci and thereby lowered CBB cycle activity, the breakdown of the organic carbon reserve glycogen is used to replenish the metabolism of *Synechocystis* at least in the transient phase before the CCM is fully activated (Eisenhut et al., 2007). However, the view that CBB cycle and glucose catabolic routes operate separately in light-exposed cyanobacteria has been modified during the last years.

Flux analyses and physiological studies in *Synechocystis* under photomixotrophic and photoautotrophic conditions revealed that external glucose or glycogen-derived glucose are metabolized in the light by entering the CBB cycle. For this purpose, glucose catabolic pathways do not operate at their full length as in darkness but instead form glycolytic shunts that enter and fine-tune the CBB cycle (Young et al., 2011; Nakajima et al., 2014; Sharkey and Weise, 2015; Matsuda et al., 2017; Shinde et al., 2019; Makowka et al., 2020). High fluxes under photomixotrophic steady state conditions were reported for the OPP shunt, whereas fluxes via the ED shunt were either not analyzed or minor (Nakajima et al., 2014; Wan et al., 2017). It has recently been shown that the OPP and the ED shunts contribute to the pools of the CBB cycle, when cells are shifted from darkness to light and glycogen is utilized to replenish the CBB cycle for a quick start of photosynthesis (Makowka et al., 2020). Similar observations were made in the cyanobacterium *Synechococcus elongatus* 7942 during dark-light shifts. Glycogen catabolism via the OPP shunt was shown to replenish intermediates of the CBB cycle and to activate CBB cycle enzymes, probably by modulation of NADPH levels (Shinde et al., 2019). Shifts between darkness and light or HC and LC conditions are alike in that sense that redox pools and the CBB cycle get perturbed and require fine-tuning. One advantage of possessing several glycolytic pathways is the potential to modulate NADPH and ATP levels distinctly dependent on the route chosen (discussed in more detail by Makowka et al., 2020).

Basically, carbon from glycogen breakdown can enter the CBB cycle either via the OPP or glycolytic shunts. By deletion of the ED shunt, replenishment of the CBB cycle via the OPP shunt is likely promoted. As a consequence, CO_2_ which is released by Gnd in the OPP shunt might reach elevated levels in Δ*eda*. Thus, this elevated decarboxylation activity should reduce photorespiration and is consistent with the reduced amount of 2PG that was observed in Δ*eda* (see Figure 6). In addition, NADPH levels could increase in Δ*eda*, because 6 NADPH are formed in the case of the OPP shunt, if three molecules of glucose 6-phosphate regenerate three molecules of ribose 5-phosphate. The dramatically enhanced level of proline (and to a lesser extent also arginine, see Figure 8) in Δ*eda* might be a response to these elevated amounts of NADPH. Proline synthesis from glutamate requires two NADPH whereas synthesis of arginine from glutamate requires one NADPH. Proline is produced as response to stress in several organisms to maintain osmotic equilibrium and particularly redox balance, thereby mitigating the effects of ROS by the modulation of NADPH levels (Fichman et al., 2015; Zhang and Becker, 2015). Along this line, we propose that the over-accumulation of proline, a substance compatible with cellular metabolism in high concentrations, might function to consume excess amounts of NADPH accumulating in Δ*eda* under LC conditions. An earlier metabolome study that quantified several sugar phosphates during acclimation from HC to LC conditions found elevated levels of 6-phosphogluconate in the WT, which points to the involvement of OPP and/or ED shunt for LC acclimation (Eisenhut et al., 2008b). Shifts from HC to LC were furthermore accompanied by increased 2PGA/3PGA ratios, which was assumed to result from increased export of 3PGA from the CBB cycle into lower glycolysis at the phosphoglycerate mutase step (Eisenhut et al., 2008b; Huege et al., 2011; Jablonsky et al., 2016; Orf et al., 2016). This finding is again well in line with the view that glucose from glycogen supports the replenishment of the CBB cycle during HC to LC shifts that allows for an increased export of 3PGA from the CBB cycle in the direction of the TCA cycle.

The notion that the observed metabolic alterations of Δ*eda* are rather based on regulatory aspects instead of missing fluxes is further supported by the fact that we observed much fewer alterations between *Synechocystis* WT and the mutant Δ*zwf* than with Δ*eda* under the different Ci conditions. The defect of Δ*zwf* is upstream of the divergence of the OPP pathway and the ED glycolytic route. In the previous metabolic scenario presented by Chen et al. (2016) a second route from glucose to 6-phosphogluconate was indicated, which circumvents glucose 6-phosphate and thus Zwf. Preliminary attempts to verify this route failed, therefore, from these observations we can rule out that a such route exists in *Synechocystis* (unpublished results of team Gutekunst). In the absence of such a bypass, the entire carbon flux from glycogen into the OPP and the ED pathways needs to include Zwf. Because we observed comparable changes in the mutant Δ*zwf* and the mutant Δ*gnd*, the latter being specifically blocked in the OPP pathway but not in the glycolytic ED route, we conclude that the observed changes in the mutant Δ*eda* are rather based on regulatory effects than changed carbon flux due to the absence of Eda. It has been also shown that in Δ*zwf* the replenishment of the CBB cycle via glycogen involves another glycolytic shunt than the ED pathway, namely the PGI shunt (for details see Makowka et al., 2020).

Collectively, our data show that deletion of *eda* deteriorates acclimation to fluctuating Ci conditions and furthermore has dramatic effects on metabolic profiles. The most prominent changes in Δ*eda* in comparison to the WT are enhanced glycogen levels under HC conditions, slowed glycogen consumption under LC conditions, retarded reactivation of growth upon shift to HC, and a dramatic accumulation of proline. Because proline is a well-known metabolite that accumulates under several stress conditions, we assume that the absence of Eda results in imbalances of redox homeostasis. In line with earlier observations (Doello et al., 2018; Makowka et al., 2020) our study provides new evidence that Eda is of importance under fluctuating conditions rather than at metabolic steady states by fine-tuning the central carbon metabolism on a regulatory level.

## Data Availability Statement

The original contributions presented in the study are included in the article/Supplementary Material, further inquiries can be directed to the corresponding author.

## Author Contributions

MH and KG designed the work and wrote the manuscript with input from all co-authors. SL, AM, and KM carried out most of the experiments and evaluated the data together with MH and KG. All authors contributed to the article and approved the submitted version.

## Conflict of Interest

The authors declare that the research was conducted in the absence of any commercial or financial relationships that could be construed as a potential conflict of interest.

## Publisher’s Note

All claims expressed in this article are solely those of the authors and do not necessarily represent those of their affiliated organizations, or those of the publisher, the editors and the reviewers. Any product that may be evaluated in this article, or claim that may be made by its manufacturer, is not guaranteed or endorsed by the publisher.
