# Supplementary material for: The Entner-Doudoroff Pathway Contributes to Glycogen Breakdown During High to Low CO2 Shifts in the Cyanobacterium Synechocystis sp. PCC 6803
Source: Front Plant Sci. 2021 Dec 9;12:787943. doi: 10.3389/fpls.2021.787943 (PMC8698341; doi:10.3389/fpls.2021.787943)
Supplement: Supplementary file 1 [file Table_1.DOCX]

**Supplementary data, Table S1 for the manuscript**

**The Entner-Doudoroff pathway contributes to glycogen breakdown during high to low CO_2_ shifts in the cyanobacterium *Synechocystis* sp. PCC 6803**

Stefan Lucius^1^, Alexander Makowka^2^, Klaudia Michl^1^, Kirstin Gutekunst^2,3^, Martin Hagemann^1,4^

1 - Institute of Biosciences, Department of Plant Physiology, University of Rostock, D-18059 Rostock, Germany

2 – Department of Biology, Botanical Institute, Christian-Albrechts-University, D-24118 Kiel, Germany

3 – Department of Molecular Plant Physiology, Bioenergetics in Photoautotrophs, University of Kassel, D-34132 Kassel, Germany

4 – Interdisciplinary Faculty, Department Life, Light and Matter, University of Rostock, D-18059 Rostock, Germany

**Supplementary Table S1:** Metabolite LC-MS data as fold changes related to respective WT HC pre-shift (set to 1) data (n = 3). Significant differences (Student’s t test p ≤ 0.05) of mutant data relative to WT data of the respective sampling points are marked in red.

| **metabolite** | **strain** | **HC pre** | **Std Err** | **LC 1h** | **Std Err** | **LC 2h** | **Std Err** | **LC 3h** | **Std Err** | **LC 24h** | **Std Err** | **HC 1h** | **Std Err** | **HC 2h** | **Std Err** | **HC 3h** | **Std Err** | **HC 24h** | **Std Err** |
| --- | --- | --- | --- | --- | --- | --- | --- | --- | --- | --- | --- | --- | --- | --- | --- | --- | --- | --- | --- |
| AMP | WT | 1.000 | 0.000 | 0.756 | 0.021 | 0.751 | 0.045 | 0.605 | 0.080 | 0.862 | 0.110 | 0.821 | 0.173 | 0.838 | 0.095 | 0.916 | 0.156 | 1.128 | 0.190 |
|  | Δ*pfk* | 1.008 | 0.725 | 0.652 | 0.433 | 0.614 | 0.404 | 0.636 | 0.457 | 0.781 | 0.520 | 0.751 | 0.519 | 0.786 | 0.613 | 0.944 | 0.655 | 1.124 | 0.727 |
|  | Δ*gnd* | 1.016 | 0.698 | 0.651 | 0.470 | 0.601 | 0.402 | 0.696 | 0.545 | 0.608 | 0.421 | 0.759 | 0.564 | 0.901 | 0.683 | 0.941 | 0.649 | 1.028 | 0.486 |
|  | Δ*eda* | 1.562 | 0.441 | 0.950 | 0.374 | 0.870 | 0.284 | 0.947 | 0.515 | 0.986 | 0.297 | 1.242 | 0.487 | 1.169 | 0.474 | 1.111 | 0.392 | 1.261 | 0.506 |
|  | Δ*zwf* | 1.276 | 0.985 | 0.542 | 0.305 | 0.591 | 0.380 | 0.537 | 0.383 | 0.867 | 0.631 | 0.894 | 0.696 | 0.925 | 0.737 | 1.156 | 0.874 | 1.039 | 0.651 |
| Alanine | WT | 1.000 | 0.000 | 1.123 | 0.141 | 0.990 | 0.152 | 0.808 | 0.163 | 0.757 | 0.115 | 0.764 | 0.156 | 0.589 | 0.100 | 0.620 | 0.112 | 0.771 | 0.222 |
|  | Δ*pfk* | 1.254 | 0.234 | 1.320 | 0.211 | 1.741 | 0.546 | 1.728 | 0.556 | 0.949 | 0.015 | 0.663 | 0.195 | 0.449 | 0.013 | 0.361 | 0.156 | 0.675 | 0.259 |
|  | Δ*gnd* | 1.026 | 0.300 | 1.136 | 0.013 | 1.036 | 0.018 | 1.013 | 0.022 | 0.571 | 0.105 | 0.570 | 0.012 | 0.573 | 0.023 | 0.668 | 0.070 | 0.751 | 0.056 |
|  | Δ*eda* | 2.377 | 0.629 | 2.151 | 0.539 | 2.202 | 0.642 | 1.969 | 0.405 | 1.056 | 0.364 | 0.849 | 0.407 | 0.831 | 0.395 | 0.830 | 0.377 | 1.070 | 0.470 |
|  | Δ*zwf* | 1.301 | 0.317 | 1.838 | 0.518 | 1.865 | 0.515 | 1.855 | 0.518 | 1.376 | 0.114 | 0.684 | 0.147 | 0.712 | 0.124 | 0.836 | 0.190 | 1.006 | 0.227 |
| Arginine | WT | 1.000 | 0.000 | 4.313 | 0.822 | 2.082 | 0.486 | 1.538 | 0.512 | 1.108 | 0.337 | 0.585 | 0.185 | 0.817 | 0.335 | 0.607 | 0.092 | 0.822 | 0.168 |
|  | Δ*pfk* | 1.639 | 0.638 | 5.055 | 2.144 | 2.957 | 0.759 | 2.934 | 1.118 | 1.445 | 0.804 | 0.399 | 0.151 | 1.028 | 0.442 | 2.250 | 1.163 | 1.673 | 0.848 |
|  | Δ*gnd* | 1.735 | 0.626 | 7.213 | 3.677 | 4.025 | 1.880 | 3.067 | 1.746 | 0.872 | 0.375 | 0.838 | 0.410 | 0.915 | 0.418 | 1.081 | 0.323 | 1.617 | 0.829 |
|  | Δ*eda* | 4.569 | 1.120 | 8.838 | 0.318 | 7.013 | 1.010 | 6.601 | 1.942 | 4.911 | 2.296 | 4.043 | 1.979 | 3.869 | 1.798 | 3.739 | 1.712 | 2.463 | 0.584 |
|  | Δ*zwf* | 2.219 | 0.674 | 4.977 | 2.011 | 3.259 | 0.441 | 3.030 | 0.810 | 1.541 | 0.754 | 0.361 | 0.188 | 0.408 | 0.184 | 0.633 | 0.089 | 1.835 | 0.770 |
| Asparagine | WT | 1.000 | 0.000 | 3.294 | 0.348 | 2.958 | 0.663 | 2.454 | 0.461 | 2.119 | 0.605 | 0.873 | 0.231 | 0.755 | 0.114 | 0.763 | 0.057 | 0.797 | 0.148 |
|  | Δ*pfk* | 1.688 | 0.031 | 5.850 | 0.744 | 5.916 | 0.116 | 7.154 | 0.317 | 4.114 | 0.880 | 0.592 | 0.148 | 0.949 | 0.125 | 1.091 | 0.080 | 1.153 | 0.134 |
|  | Δ*gnd* | 1.191 | 0.159 | 3.481 | 0.652 | 3.115 | 0.597 | 3.596 | 0.577 | 1.756 | 0.114 | 0.668 | 0.075 | 0.751 | 0.085 | 0.846 | 0.011 | 0.823 | 0.141 |
|  | Δ*eda* | 1.214 | 0.258 | 1.754 | 0.581 | 2.346 | 0.998 | 3.316 | 0.993 | 1.415 | 0.447 | 0.534 | 0.093 | 0.624 | 0.065 | 0.615 | 0.068 | 0.813 | 0.097 |
|  | Δ*zwf* | 2.726 | 0.486 | 7.790 | 0.025 | 7.826 | 0.870 | 9.098 | 0.515 | 5.238 | 0.072 | 0.774 | 0.451 | 1.192 | 0.092 | 1.167 | 0.258 | 1.667 | 0.073 |
| Aspartic Acid | WT | 1.000 | 0.000 | 0.970 | 0.210 | 1.021 | 0.232 | 0.776 | 0.171 | 0.800 | 0.252 | 0.638 | 0.186 | 0.677 | 0.261 | 0.805 | 0.275 | 0.849 | 0.230 |
|  | Δ*pfk* | 1.259 | 0.095 | 1.030 | 0.203 | 1.301 | 0.053 | 1.621 | 0.042 | 0.803 | 0.185 | 1.451 | 0.299 | 0.790 | 0.351 | 0.664 | 0.178 | 0.677 | 0.131 |
|  | Δ*gnd* | 0.327 | 0.013 | 0.466 | 0.004 | 0.360 | 0.073 | 0.373 | 0.102 | 0.089 | 0.015 | 0.550 | 0.181 | 0.620 | 0.050 | 0.794 | 0.153 | 0.183 | 0.091 |
|  | Δ*eda* | 1.008 | 0.417 | 0.757 | 0.014 | 0.928 | 0.104 | 1.054 | 0.275 | 0.446 | 0.072 | 0.469 | 0.048 | 0.564 | 0.029 | 0.635 | 0.071 | 0.523 | 0.065 |
|  | Δ*zwf* | 0.828 | 0.179 | 1.056 | 0.018 | 1.239 | 0.258 | 1.521 | 0.026 | 0.606 | 0.001 | 0.979 | 0.166 | 1.031 | 0.019 | 0.936 | 0.359 | 0.500 | 0.144 |
| Citric Acid | WT | 1.000 | 0.000 | 1.079 | 0.232 | 1.048 | 0.193 | 0.892 | 0.200 | 1.089 | 0.210 | 0.926 | 0.134 | 1.264 | 0.412 | 1.265 | 0.314 | 1.022 | 0.154 |
|  | Δ*pfk* | 1.771 | 0.719 | 1.332 | 0.552 | 1.341 | 0.456 | 1.335 | 0.595 | 1.467 | 0.633 | 1.320 | 0.490 | 1.357 | 0.584 | 1.289 | 0.390 | 1.477 | 0.436 |
|  | Δ*gnd* | 1.289 | 0.469 | 1.131 | 0.527 | 1.105 | 0.490 | 1.072 | 0.566 | 0.752 | 0.263 | 0.857 | 0.250 | 0.928 | 0.331 | 0.997 | 0.260 | 1.137 | 0.320 |
|  | Δ*eda* | 1.395 | 0.334 | 0.908 | 0.280 | 0.852 | 0.252 | 0.867 | 0.335 | 0.629 | 0.170 | 0.672 | 0.217 | 0.814 | 0.270 | 0.772 | 0.232 | 0.915 | 0.278 |
|  | Δ*zwf* | 1.967 | 0.718 | 1.334 | 0.323 | 1.440 | 0.409 | 1.189 | 0.398 | 1.387 | 0.565 | 1.310 | 0.393 | 1.373 | 0.461 | 1.561 | 0.605 | 1.550 | 0.532 |

| **metabolite** | **strain** | **HC pre** | **Std Err** | **LC 1h** | **Std Err** | **LC 2h** | **Std Err** | **LC 3h** | **Std Err** | **LC 24h** | **Std Err** | **HC 1h** | **Std Err** | **HC 2h** | **Std Err** | **HC 3h** | **Std Err** | **HC 24h** | **Std Err** |
| --- | --- | --- | --- | --- | --- | --- | --- | --- | --- | --- | --- | --- | --- | --- | --- | --- | --- | --- | --- |
| Glutamic Acid | WT | 1.000 | 0.000 | 0.428 | 0.053 | 0.486 | 0.052 | 0.399 | 0.075 | 0.835 | 0.280 | 0.697 | 0.214 | 0.659 | 0.143 | 0.709 | 0.165 | 0.996 | 0.067 |
|  | Δ*pfk* | 1.560 | 0.009 | 0.662 | 0.109 | 0.736 | 0.087 | 0.815 | 0.023 | 1.389 | 0.233 | 0.884 | 0.068 | 0.973 | 0.048 | 1.086 | 0.133 | 1.444 | 0.014 |
|  | Δ*gnd* | 1.302 | 0.063 | 0.405 | 0.059 | 0.490 | 0.024 | 0.476 | 0.045 | 0.420 | 0.015 | 0.538 | 0.035 | 0.556 | 0.013 | 0.578 | 0.014 | 1.026 | 0.124 |
|  | Δ*eda* | 0.736 | 0.119 | 0.422 | 0.089 | 0.499 | 0.066 | 0.601 | 0.132 | 0.603 | 0.169 | 0.799 | 0.205 | 0.838 | 0.230 | 0.792 | 0.240 | 0.701 | 0.307 |
|  | Δ*zwf* | 1.763 | 0.173 | 0.911 | 0.090 | 0.896 | 0.107 | 1.019 | 0.089 | 1.506 | 0.249 | 1.156 | 0.047 | 1.163 | 0.029 | 1.343 | 0.001 | 1.453 | 0.106 |
| Glutamine | WT | 1.000 | 0.000 | 1.265 | 0.031 | 1.481 | 0.287 | 0.787 | 0.159 | 0.819 | 0.296 | 0.996 | 0.242 | 0.777 | 0.232 | 0.868 | 0.216 | 0.474 | 0.144 |
|  | Δ*pfk* | 1.448 | 0.148 | 0.767 | 0.332 | 1.086 | 0.289 | 1.232 | 0.128 | 0.938 | 0.367 | 2.609 | 0.543 | 1.345 | 0.905 | 0.718 | 0.048 | 0.659 | 0.222 |
|  | Δ*gnd* | 0.818 | 0.221 | 0.705 | 0.240 | 0.607 | 0.195 | 0.570 | 0.066 | 0.286 | 0.061 | 0.912 | 0.230 | 0.981 | 0.074 | 0.970 | 0.043 | 0.314 | 0.075 |
|  | Δ*eda* | 1.450 | 0.381 | 1.864 | 0.428 | 1.799 | 0.364 | 1.328 | 0.353 | 0.372 | 0.035 | 0.863 | 0.286 | 0.955 | 0.219 | 1.088 | 0.126 | 0.528 | 0.100 |
|  | Δ*zwf* | 0.859 | 0.128 | 0.739 | 0.308 | 1.316 | 0.706 | 1.198 | 0.192 | 0.579 | 0.128 | 1.993 | 0.033 | 2.456 | 0.053 | 2.018 | 1.279 | 0.322 | 0.089 |
| Glycine | WT | 1.000 | 0.000 | 1.015 | 0.140 | 0.955 | 0.134 | 0.727 | 0.200 | 0.940 | 0.221 | 0.663 | 0.195 | 0.890 | 0.182 | 0.769 | 0.132 | 0.758 | 0.146 |
|  | Δ*pfk* | 1.495 | 0.039 | 1.109 | 0.135 | 1.228 | 0.148 | 1.504 | 0.332 | 1.412 | 0.807 | 0.779 | 0.084 | 0.818 | 0.169 | 0.623 | 0.060 | 0.856 | 0.042 |
|  | Δ*gnd* | 1.075 | 0.148 | 1.106 | 0.323 | 1.017 | 0.327 | 0.726 | 0.178 | 0.482 | 0.076 | 0.652 | 0.040 | 0.659 | 0.100 | 0.683 | 0.010 | 0.682 | 0.113 |
|  | Δ*eda* | 1.811 | 0.376 | 1.532 | 0.202 | 1.743 | 0.402 | 1.369 | 0.198 | 1.173 | 0.167 | 1.028 | 0.272 | 1.111 | 0.283 | 1.123 | 0.272 | 1.028 | 0.203 |
|  | Δ*zwf* | 1.182 | 0.036 | 0.968 | 0.009 | 1.092 | 0.125 | 1.143 | 0.070 | 0.808 | 0.188 | 0.563 | 0.038 | 0.654 | 0.064 | 0.706 | 0.071 | 0.734 | 0.035 |
| Isocitric Acid | WT | 1.000 | 0.000 | 1.075 | 0.238 | 1.036 | 0.195 | 0.893 | 0.205 | 1.094 | 0.220 | 0.924 | 0.144 | 1.279 | 0.430 | 1.261 | 0.323 | 1.021 | 0.168 |
|  | Δ*pfk* | 1.737 | 0.733 | 1.312 | 0.578 | 1.323 | 0.490 | 1.321 | 0.612 | 1.453 | 0.665 | 1.304 | 0.516 | 1.337 | 0.609 | 1.265 | 0.418 | 1.458 | 0.485 |
|  | Δ*gnd* | 1.287 | 0.498 | 1.108 | 0.535 | 1.095 | 0.513 | 1.056 | 0.577 | 0.739 | 0.272 | 0.843 | 0.268 | 0.920 | 0.356 | 0.981 | 0.284 | 1.130 | 0.358 |
|  | Δ*eda* | 1.364 | 0.336 | 0.896 | 0.285 | 0.830 | 0.262 | 0.849 | 0.328 | 0.612 | 0.175 | 0.662 | 0.217 | 0.804 | 0.282 | 0.761 | 0.239 | 0.890 | 0.279 |
|  | Δ*zwf* | 1.970 | 0.780 | 1.326 | 0.358 | 1.434 | 0.447 | 1.176 | 0.436 | 1.363 | 0.593 | 1.285 | 0.416 | 1.346 | 0.487 | 1.555 | 0.647 | 1.551 | 0.592 |
| Lactic Acid | WT | 1.000 | 0.000 | 1.391 | 0.183 | 0.820 | 0.096 | 0.942 | 0.119 | 1.079 | 0.134 | 1.403 | 0.425 | 0.938 | 0.178 | 1.042 | 0.126 | 0.474 | 0.131 |
|  | Δ*pfk* | 1.271 | 0.961 | 1.001 | 0.460 | 0.863 | 0.384 | 1.116 | 0.602 | 1.092 | 0.621 | 0.534 | 0.218 | 0.745 | 0.606 | 0.720 | 0.502 | 0.243 | 0.072 |
|  | Δ*gnd* | 0.919 | 0.345 | 1.125 | 0.426 | 1.673 | 0.934 | 1.259 | 0.749 | 1.040 | 0.528 | 0.576 | 0.252 | 1.032 | 0.709 | 0.650 | 0.345 | 0.295 | 0.212 |
|  | Δ*eda* | 1.100 | 0.151 | 1.032 | 0.272 | 0.972 | 0.217 | 1.127 | 0.481 | 0.853 | 0.135 | 0.644 | 0.209 | 0.622 | 0.197 | 0.522 | 0.154 | 0.475 | 0.187 |
|  | Δ*zwf* | 1.140 | 0.742 | 0.837 | 0.496 | 1.015 | 0.618 | 1.038 | 0.593 | 0.698 | 0.420 | 0.784 | 0.510 | 0.595 | 0.406 | 1.015 | 0.796 | 0.454 | 0.385 |
| Lysine | WT | 1.000 | 0.000 | 1.205 | 0.132 | 1.377 | 0.324 | 0.819 | 0.221 | 1.092 | 0.312 | 0.592 | 0.070 | 0.523 | 0.098 | 0.632 | 0.173 | 0.620 | 0.166 |
|  | Δ*pfk* | 1.510 | 0.109 | 1.614 | 0.496 | 2.274 | 0.878 | 2.179 | 0.792 | 2.838 | 1.489 | 2.508 | 0.682 | 1.400 | 0.860 | 0.503 | 0.174 | 0.610 | 0.269 |
|  | Δ*gnd* | 1.054 | 0.004 | 1.217 | 0.229 | 1.435 | 0.618 | 1.201 | 0.555 | 0.807 | 0.446 | 0.604 | 0.097 | 0.580 | 0.341 | 0.565 | 0.367 | 0.288 | 0.100 |
|  | Δ*eda* | 1.122 | 0.124 | 1.287 | 0.234 | 1.470 | 0.424 | 1.346 | 0.487 | 0.762 | 0.323 | 0.972 | 0.196 | 0.881 | 0.225 | 0.846 | 0.231 | 0.748 | 0.223 |
|  | Δ*zwf* | 1.254 | 0.268 | 1.850 | 0.800 | 2.574 | 0.546 | 2.194 | 0.787 | 2.173 | 1.448 | 1.805 | 0.252 | 1.509 | 1.046 | 1.779 | 1.548 | 0.535 | 0.089 |
| Malic Acid | WT | 1.000 | 0.000 | 2.051 | 1.065 | 0.883 | 0.279 | 1.852 | 0.885 | 1.588 | 0.610 | 1.634 | 0.483 | 1.826 | 0.314 | 1.711 | 0.131 | 1.712 | 0.244 |
|  | Δ*pfk* | 1.581 | 0.046 | 1.741 | 0.266 | 0.802 | 0.360 | 1.745 | 0.068 | 1.660 | 0.373 | 1.671 | 0.665 | 4.512 | 2.994 | 1.861 | 0.067 | 2.451 | 1.176 |
|  | Δ*gnd* | 2.898 | 0.739 | 2.138 | 0.438 | 1.763 | 0.538 | 1.768 | 0.107 | 1.612 | 0.980 | 1.731 | 0.509 | 1.537 | 0.308 | 1.265 | 0.208 | 3.504 | 1.296 |
|  | Δ*eda* | 1.807 | 0.796 | 1.827 | 1.066 | 1.756 | 1.170 | 0.721 | 0.145 | 2.460 | 0.945 | 2.154 | 0.493 | 2.436 | 0.548 | 2.593 | 0.716 | 1.680 | 0.579 |
|  | Δ*zwf* | 1.575 | 0.016 | 0.897 | 0.277 | 0.390 | 0.123 | 1.183 | 0.289 | 2.198 | 1.192 | 1.361 | 0.537 | 1.386 | 0.306 | 2.225 | 0.808 | 3.681 | 0.960 |

| **metabolite** | **strain** | **HC pre** | **Std Err** | **LC 1h** | **Std Err** | **LC 2h** | **Std Err** | **LC 3h** | **Std Err** | **LC 24h** | **Std Err** | **HC 1h** | **Std Err** | **HC 2h** | **Std Err** | **HC 3h** | **Std Err** | **HC 24h** | **Std Err** |
| --- | --- | --- | --- | --- | --- | --- | --- | --- | --- | --- | --- | --- | --- | --- | --- | --- | --- | --- | --- |
| Methionine | WT | 1.000 | 0.000 | 0.468 | 0.090 | 0.419 | 0.128 | 0.474 | 0.306 | 0.534 | 0.325 | 0.601 | 0.216 | 0.713 | 0.201 | 0.742 | 0.246 | 0.859 | 0.284 |
|  | Δ*pfk* | 1.029 | 0.189 | 0.687 | 0.043 | 0.804 | 0.309 | 0.512 | 0.075 | 0.681 | 0.579 | 0.673 | 0.020 | 0.696 | 0.025 | 0.612 | 0.519 | 0.720 | 0.401 |
|  | Δ*gnd* | 1.105 | 0.044 | 0.412 | 0.129 | 0.502 | 0.049 | 0.348 | 0.103 | 0.197 | 0.121 | 0.467 | 0.038 | 0.434 | 0.011 | 0.260 | 0.185 | 0.525 | 0.366 |
|  | Δ*eda* | 1.369 | 0.456 | 0.449 | 0.123 | 0.621 | 0.201 | 0.507 | 0.093 | 0.386 | 0.099 | 0.514 | 0.164 | 0.540 | 0.163 | 0.440 | 0.165 | 0.543 | 0.211 |
|  | Δ*zwf* | 1.370 | 0.113 | 0.678 | 0.074 | 1.066 | 0.081 | 0.506 | 0.134 | 0.730 | 0.537 | 0.777 | 0.074 | 0.923 | 0.009 | 0.589 | 0.487 | 0.669 | 0.543 |
| 3-Phospho- glycerate | WT | 1.000 | 0.000 | 4.668 | 1.118 | 4.009 | 1.302 | 4.079 | 1.513 | 5.876 | 2.026 | 2.849 | 1.026 | 3.749 | 2.000 | 3.367 | 1.733 | 1.830 | 0.948 |
|  | Δ*pfk* | 2.027 | 0.420 | 11.649 | 4.461 | 7.041 | 2.180 | 9.830 | 3.956 | 9.334 | 4.090 | 4.546 | 2.452 | 4.170 | 2.598 | 3.242 | 1.896 | 1.625 | 1.248 |
|  | Δ*gnd* | 1.529 | 0.752 | 9.025 | 5.841 | 6.717 | 4.381 | 7.123 | 4.987 | 7.504 | 4.254 | 0.749 | 0.388 | 0.835 | 0.518 | 0.711 | 0.209 | 0.371 | 0.203 |
|  | Δ*eda* | 0.963 | 0.397 | 2.845 | 1.281 | 2.646 | 1.018 | 3.039 | 1.409 | 1.810 | 0.582 | 0.553 | 0.228 | 0.592 | 0.269 | 0.676 | 0.367 | 0.452 | 0.256 |
|  | Δ*zwf* | 1.381 | 0.415 | 6.024 | 2.570 | 3.983 | 1.705 | 6.598 | 3.160 | 7.404 | 3.892 | 3.027 | 2.186 | 2.458 | 1.948 | 2.836 | 2.257 | 0.524 | 0.422 |
| 2-Phospho- glycolate | WT | 1.000 | 0.000 | 12.852 | 6.329 | 15.519 | 9.308 | 10.170 | 5.556 | 7.721 | 2.828 | 1.295 | 0.222 | 1.184 | 0.283 | 1.349 | 0.272 | 0.779 | 0.107 |
|  | Δ*pfk* | 2.477 | 0.124 | 16.894 | 13.856 | 22.863 | 20.010 | 21.737 | 17.101 | 4.484 | 1.735 | 1.925 | 0.122 | 2.036 | 0.086 | 2.002 | 0.009 | 0.951 | 0.281 |
|  | Δ*gnd* | 2.681 | 0.018 | 19.220 | 15.443 | 19.493 | 16.672 | 18.130 | 14.736 | 11.491 | 8.246 | 1.143 | 0.482 | 1.256 | 0.756 | 1.552 | 0.935 | 0.519 | 0.146 |
|  | Δ*eda* | 1.272 | 0.713 | 4.482 | 2.002 | 5.105 | 2.614 | 4.489 | 2.047 | 2.199 | 0.451 | 1.262 | 0.448 | 1.442 | 0.571 | 1.169 | 0.416 | 0.929 | 0.371 |
|  | Δ*zwf* | 2.073 | 0.104 | 16.318 | 12.411 | 20.864 | 17.562 | 19.796 | 15.396 | 5.249 | 2.618 | 1.633 | 0.349 | 2.129 | 0.275 | 2.299 | 0.199 | 0.918 | 0.101 |
| Proline | WT | 1.000 | 0.000 | 3.399 | 0.555 | 1.719 | 0.473 | 1.103 | 0.389 | 0.571 | 0.132 | 0.616 | 0.251 | 0.545 | 0.130 | 0.524 | 0.026 | 0.552 | 0.057 |
|  | Δ*pfk* | 1.741 | 0.625 | 7.760 | 2.407 | 2.429 | 0.653 | 2.620 | 0.663 | 1.466 | 0.857 | 0.626 | 0.144 | 1.702 | 0.814 | 2.050 | 1.105 | 0.721 | 0.042 |
|  | Δ*gnd* | 3.146 | 0.522 | 5.558 | 0.656 | 3.221 | 1.387 | 2.468 | 1.014 | 0.700 | 0.179 | 0.395 | 0.101 | 0.462 | 0.164 | 0.567 | 0.065 | 0.832 | 0.231 |
|  | Δ*eda* | 14.984 | 3.577 | 47.585 | 22.992 | 39.858 | 26.350 | 35.662 | 27.309 | 8.794 | 3.638 | 3.009 | 0.951 | 2.456 | 0.733 | 2.011 | 0.529 | 1.559 | 0.303 |
|  | Δ*zwf* | 2.835 | 1.319 | 8.608 | 2.405 | 3.466 | 0.067 | 4.076 | 1.041 | 1.176 | 0.352 | 0.574 | 0.181 | 0.656 | 0.216 | 0.842 | 0.100 | 0.878 | 0.078 |
| Serine | WT | 1.000 | 0.000 | 2.104 | 0.749 | 1.601 | 0.389 | 1.149 | 0.236 | 1.534 | 0.421 | 1.738 | 0.983 | 0.886 | 0.123 | 0.856 | 0.141 | 0.753 | 0.221 |
|  | Δ*pfk* | 1.561 | 0.091 | 2.626 | 0.062 | 3.250 | 0.731 | 3.292 | 0.482 | 2.600 | 0.644 | 1.204 | 0.310 | 0.953 | 0.008 | 0.846 | 0.228 | 0.739 | 0.125 |
|  | Δ*gnd* | 1.478 | 0.180 | 1.544 | 0.041 | 1.538 | 0.086 | 1.359 | 0.057 | 0.962 | 0.051 | 0.836 | 0.123 | 0.779 | 0.026 | 0.852 | 0.137 | 0.526 | 0.124 |
|  | Δ*eda* | 2.578 | 0.842 | 1.920 | 0.530 | 2.039 | 0.590 | 3.345 | 1.244 | 1.040 | 0.226 | 0.742 | 0.148 | 0.791 | 0.153 | 0.671 | 0.020 | 1.307 | 0.651 |
|  | Δ*zwf* | 1.154 | 0.213 | 2.825 | 0.511 | 3.212 | 0.268 | 3.533 | 0.593 | 2.141 | 0.041 | 0.959 | 0.226 | 1.017 | 0.139 | 1.087 | 0.053 | 0.833 | 0.019 |
| Succinic Acid | WT | 1.000 | 0.000 | 1.211 | 0.241 | 1.299 | 0.271 | 1.502 | 0.433 | 1.730 | 0.610 | 2.125 | 1.391 | 1.263 | 0.419 | 1.328 | 0.335 | 2.896 | 0.552 |
|  | Δ*pfk* | 2.095 | 0.563 | 2.361 | 0.451 | 1.502 | 0.056 | 2.590 | 0.918 | 2.949 | 1.158 | 1.696 | 0.083 | 4.535 | 2.436 | 3.737 | 1.527 | 3.910 | 1.358 |
|  | Δ*gnd* | 5.519 | 0.489 | 3.246 | 0.888 | 3.263 | 0.495 | 4.459 | 1.214 | 5.842 | 1.161 | 2.154 | 0.769 | 2.387 | 0.476 | 2.206 | 0.593 | 7.019 | 0.388 |
|  | Δ*eda* | 1.067 | 0.373 | 0.895 | 0.402 | 0.852 | 0.253 | 1.377 | 0.705 | 1.307 | 0.297 | 1.041 | 0.279 | 1.045 | 0.240 | 1.125 | 0.253 | 1.314 | 0.724 |
|  | Δ*zwf* | 4.105 | 1.053 | 3.188 | 0.662 | 2.266 | 0.181 | 4.473 | 0.788 | 5.883 | 2.392 | 2.394 | 0.693 | 2.247 | 0.692 | 3.149 | 0.084 | 7.344 | 1.553 |
| Threonine | WT | 1.000 | 0.000 | 1.179 | 0.145 | 1.088 | 0.128 | 0.868 | 0.119 | 1.023 | 0.154 | 1.280 | 0.435 | 0.900 | 0.072 | 0.928 | 0.094 | 0.896 | 0.087 |
|  | Δ*pfk* | 1.543 | 0.044 | 1.786 | 0.060 | 2.007 | 0.190 | 2.139 | 0.109 | 1.727 | 0.329 | 1.447 | 0.147 | 1.229 | 0.046 | 1.118 | 0.281 | 1.358 | 0.149 |
|  | Δ*gnd* | 1.433 | 0.069 | 1.330 | 0.091 | 1.412 | 0.239 | 1.336 | 0.215 | 1.087 | 0.093 | 1.433 | 0.221 | 1.321 | 0.050 | 1.366 | 0.010 | 1.127 | 0.100 |
|  | Δ*eda* | 2.411 | 0.494 | 2.194 | 0.321 | 2.152 | 0.292 | 2.626 | 0.566 | 1.226 | 0.221 | 1.030 | 0.124 | 1.053 | 0.101 | 0.977 | 0.126 | 1.177 | 0.080 |
|  | Δ*zwf* | 1.529 | 0.134 | 1.947 | 0.208 | 2.168 | 0.245 | 2.188 | 0.150 | 1.652 | 0.134 | 1.362 | 0.007 | 1.406 | 0.019 | 1.552 | 0.090 | 1.330 | 0.085 |

| **metabolite** | **strain** | **HC pre** | **Std Err** | **LC 1h** | **Std Err** | **LC 2h** | **Std Err** | **LC 3h** | **Std Err** | **LC 24h** | **Std Err** | **HC 1h** | **Std Err** | **HC 2h** | **Std Err** | **HC 3h** | **Std Err** | **HC 24h** | **Std Err** |
| --- | --- | --- | --- | --- | --- | --- | --- | --- | --- | --- | --- | --- | --- | --- | --- | --- | --- | --- | --- |
| Tryptophan | WT | 1.000 | 0.000 | 1.785 | 0.357 | 1.105 | 0.152 | 0.779 | 0.057 | 1.007 | 0.192 | 2.070 | 0.479 | 1.873 | 0.280 | 1.827 | 0.159 | 1.117 | 0.241 |
|  | Δ*pfk* | 1.730 | 0.864 | 2.075 | 1.114 | 1.906 | 0.704 | 2.760 | 1.624 | 0.520 | 0.000 | 2.081 | 1.101 | 2.495 | 0.989 | 1.830 | 0.549 | 2.071 | 0.083 |
|  | Δ*gnd* | 1.958 | 0.778 | 2.641 | 1.262 | 2.546 | 1.265 | 2.210 | 1.046 | 1.736 | 1.162 | 1.993 | 0.979 | 2.138 | 0.949 | 2.059 | 0.458 | 1.983 | 0.969 |
|  | Δ*eda* | 4.014 | 0.886 | 3.364 | 0.565 | 3.106 | 0.632 | 2.623 | 0.746 | 2.529 | 0.584 | 2.400 | 0.531 | 2.523 | 0.515 | 2.184 | 0.487 | 2.329 | 0.606 |
|  | Δ*zwf* | 3.792 | 2.129 | 2.234 | 0.809 | 1.755 | 0.677 | 2.291 | 1.149 | 2.124 | 1.616 | 3.038 | 1.676 | 3.064 | 1.505 | 3.147 | 1.408 | 2.477 | 0.881 |
| Tyrosine | WT | 1.000 | 0.000 | 0.929 | 0.134 | 0.724 | 0.029 | 0.508 | 0.046 | 0.549 | 0.058 | 0.853 | 0.070 | 0.829 | 0.055 | 0.886 | 0.116 | 0.628 | 0.043 |
|  | Δ*pfk* | 1.211 | 0.386 | 1.159 | 0.298 | 0.945 | 0.233 | 0.946 | 0.379 | 1.563 | 1.190 | 1.324 | 0.466 | 1.282 | 0.387 | 0.989 | 0.152 | 0.700 | 0.001 |
|  | Δ*gnd* | 1.570 | 0.400 | 1.367 | 0.559 | 1.430 | 0.621 | 1.310 | 0.643 | 0.860 | 0.392 | 1.357 | 0.531 | 1.400 | 0.492 | 1.525 | 0.377 | 0.958 | 0.002 |
|  | Δ*eda* | 2.798 | 0.215 | 2.769 | 0.488 | 2.463 | 0.486 | 2.455 | 0.687 | 1.497 | 0.387 | 1.422 | 0.371 | 1.444 | 0.356 | 1.343 | 0.309 | 1.438 | 0.412 |
|  | Δ*zwf* | 1.730 | 0.713 | 1.137 | 0.229 | 0.951 | 0.267 | 0.808 | 0.274 | 0.686 | 0.279 | 1.192 | 0.506 | 1.174 | 0.380 | 1.384 | 0.334 | 0.783 | 0.013 |
| Valine | WT | 1.000 | 0.000 | 2.800 | 0.468 | 2.427 | 0.413 | 1.892 | 0.286 | 1.805 | 0.329 | 1.388 | 0.462 | 1.063 | 0.204 | 1.260 | 0.251 | 1.212 | 0.332 |
|  | Δ*pfk* | 1.550 | 0.190 | 5.209 | 0.355 | 6.999 | 1.368 | 4.563 | 0.167 | 3.606 | 1.330 | 1.459 | 0.003 | 1.143 | 0.207 | 1.033 | 0.298 | 1.249 | 0.076 |
|  | Δ*gnd* | 1.515 | 0.147 | 2.385 | 0.246 | 2.393 | 0.503 | 2.271 | 0.532 | 0.978 | 0.171 | 1.484 | 0.366 | 1.568 | 0.543 | 1.729 | 0.403 | 1.526 | 0.598 |
|  | Δ*eda* | 2.839 | 0.458 | 2.781 | 0.767 | 4.403 | 1.687 | 4.127 | 0.596 | 2.322 | 0.858 | 1.351 | 0.388 | 1.440 | 0.415 | 1.432 | 0.408 | 2.488 | 0.843 |
|  | Δ*zwf* | 1.558 | 0.256 | 4.979 | 0.208 | 7.602 | 0.684 | 5.414 | 0.264 | 3.292 | 0.725 | 1.310 | 0.248 | 1.326 | 0.271 | 1.444 | 0.306 | 1.742 | 0.060 |
